# Reasons for a Lower Rate of Epidural Anesthesia During Birth for Immigrant Women in the Eyes of Medical Staff: A Mixed-Methods Analysis

**DOI:** 10.1007/s10903-022-01357-1

**Published:** 2022-04-07

**Authors:** Vera Seidel, Claudia Großkreutz, Burcu Gürbüz, Wolfgang Henrich, Rebecca C. Rancourt, Theda Borde, Matthias David

**Affiliations:** 1 Clinic of Obstetrics, Charité – Universitätsmedizin Berlin, Berlin, Germany; 2 Berlin Institute of Health (BIH), Berlin, Germany; 3 Division of ‘Experimental Obstetrics’, Clinic of Obstetrics, Charité – Universitätsmedizin, Berlin, Germany; 4 Alice Salomon Hochschule Berlin, Berlin, Germany; 5 Clinic of Gynecology, Charité – Universitätsmedizin Berlin, Berlin, Germany

**Keywords:** Epidural anesthesia, Obstetrics, Immigrant health, Birth, Labour

## Abstract

Various studies have shown that immigrant women in comparison to non-immigrant women of the same parity have lower rates of epidural anesthesia (EDA). Data from two studies on immigrant obstetric care in Berlin, Germany were analyzed to answer the following question: What reasons do the medical staff see for the lower rate of EDA in immigrant women? Between May and August 2017, 34 interviews with obstetricians and midwives in four obstetric clinics in Berlin were conducted on the topic of obstetric care for immigrant women. After anonymizing the more than 20 h of interview material, transcripts were coded with MaxQDa and analyzed according to the qualitative content analysis.The quantitative data is from an online survey conducted between May and October 2017, in all but one obstetric clinic in Berlin with obstetricians and midwives. Regarding the research question, 121 questionnaires could be analyzed. In the online survey, (multiple answers were possible), the top reason for a lower rate of EDA given was mostly fear on the part of the immigrant women (64%). A language barrier, which results in logistic and time constrictions, is mentioned as the second most frequent reason (50%). The explorative analysis of the interviews shows that doctors and midwives regard cultural aspects such as different expectations on the birth experience as a reason for a lower EDA rate. Furthermore, within the medical staff the impression persists that in some cases the companion decides on the behalf of the patient about the application of an EDA, which from time to time is against the wish of the immigrant woman giving birth. In the view of the medical staff, the reasons for a lower rate of EDA during birth for immigrant women were varied. On one side, this is attributed to the wishes of the respective women (“demand”) but on the other side this can be attributed to the health care system (“supply”). In the case of a language barrier, the “supply” and the access of EDA for immigrant women is limited and can be then shifted to the German-speaking companion to make a decision regarding EDA (“structural deprivation of self-determination”).

## Introduction

International migration is a growing phenomenon. There are several studies investigating the perinatal outcome of certain groups of immigrants in Germany which show no heightened perinatal morbidity or mortality [1–4]. While this is comforting regarding inclusion capacities of this health care system, differences are still detectable.

A study in Berlin showed that immigrant women from Turkey and Lebanon less frequently make use of epidural anesthesia (EDA) during labor [5]. This effect can also be observed in other countries. In the United Kingdom, the rate of EDA is lower for Kosovo Albanian asylum-seekers [6]. In Georgia, USA, race/ethnicity other than white/non-Hispanic was identified as a significant predictor for lower EDA rates even after controlling for age, rural–urban residence and capacity of anesthesiologists [7]. Petruschke et al. discussed two main clusters of reasons for lower EDA rates among immigrants [8]. In the case of the ‘‘supply’’: the medical staff might not pay as much attention to the expressions of pain of immigrants during the delivery or there may be language problems when providing information and explaining the options for perinatal pain control. Furthermore, giving informed consent is more difficult in the case of a language barrier leading to legal concerns on the part of the health care staff. Rather the “demand” might be a reason: immigrants seem to request EDA less often due to lack of knowledge, anxiety about side effects or because of communication problems [5].

A qualitative study in Berlin, in which immigrant women from Turkey were interviewed before giving birth, found the potential reasons for rejection of EDA was fear of long-term complications and the view that vaginal delivery with EDA is not natural. Furthermore, information on EDA is frequently obtained from a variety of sources from their social setting, in particular, by word of mouth [8]. Language barriers can lead to less information on anesthesia for labor pain during labor and/or women with limited German proficiency (LGP) cannot articulate their pain sufficiently. Studies from the USA in various medical contexts have also shown that patients with limited English proficiency when admitted to the hospital receive insufficient anesthesia [9, 10]. The main hypothesis, the authors discuss in their study, is that the way Hispanics express their pain is culturally different from the way physicians in the emergency room are used to within their own cultural group so they do not interpret the presented symptoms correctly [10].

So far, no systematic studies on the view of medical staff for the lower rate of EDA among immigrant women during labor and birth have been conducted. With our study, we wanted to answer the following question: What reasons do the medical staff see for the lower rate of EDA in immigrant women?

## Material and Methods

Data for this analysis comes from a quantitative online-survey, which was conducted between May and October 2017. Participation in this study was offered to all midwives and obstetricians (physicians during their residency to eventually become obstetricians were included in this group) in all obstetric clinics in Berlin. The staff council for only one clinic rejected participation in the study.

Approval was given by the ethics committee of the Charité – Universitätsmedizin Berlin (EA2/187/16). The survey was conducted with the software QUAMP [(Modul qSurveyor) is a webbased, modular software platform for internet-based feedback retrieval (V. 4.0.0.4).] Participants were invited via email, blackboards and by a personal introduction of the study in morning clinic meetings by first author VS and trained study assistants. Participants gave their informed consent to study participation. The data collection was anonymous. For a more detailed description of the methodology of this study see also Seidel et al. [11]. The questionnaire contained 35 multiple-choice questions and 6 open questions. In this article, we focus on the questions regarding EDA use during labor and birth. Comparison between obstetric health care professions was performed with the Fisher’s Exact Test in the program IBM© SPSS© Statistics, Version 25, © Copyright 1989, 2016 SPSS Inc., an IBM Company.

Furthermore, there was an open question in the online-questionnaire if the participants regarded other important reasons than the options given. This qualitative data received in the free text was grouped in thematic fields.

Further qualitative data was collected in an interview study. Between May and August 2017, 34 semi-structured interviews with obstetricians and midwives delivering perinatal care in four different hospitals in Berlin were conducted by the research team that consisted of two doctoral students and one physician working in obstetrics and gynecology (note: the staff was all female in this study). These interviewers had no prior contact or relation to the participants and were all previously trained in qualitative data acquisition. After introducing themselves as researchers and mentioning their respective professions, the interviewers explained the purpose of the study. Written consent was then given by the interviewees willing to participate. The interviews were audiotaped and then subsequently verbally transcribed. To ensure confidentiality, all information allowing any identification of the person or corresponding clinic was removed from the transcripts. Transcripts were checked twice (by the first and second author) to ensure the accuracy of the transcription before coding. Qualitative content analysis was performed inductively according to the steps proposed by Mayring [12]. Two researchers (first and second author) performed coding. Data analysis was initiated parallel to the conduction of interviews so that data saturation could be detected during the period of data collection [13] and research could accordingly be concluded after 34 of the initial 40 planned interviews. Analytical codes were organized into main themes und subthemes with the program MAXQDA Plus 12 (Release 12.3.1, VERBI GmbH Berlin). Coherence of coding was checked in a random sample of interview transcripts and discrepancies were discussed between the researchers to ensure the validity and the credibility of the results. A total of 12 h and 15 min of interview material was recorded. Saturation of information already occurred after 23 interviews; however, the total reached 34 as dates with the interviewees had already been scheduled.

The primary interest of the study was to elucidate which differences and similarities there are in the perinatal care between immigrant and non-immigrant women in the view of obstetric medical staff. Furthermore, the challenges for the medical staff and their coping strategies were assessed; reasons for differences in uptake of care and wishes for the future were touched on. For the purposes of this specific paper only, the data on reasons for lower EDA rates among immigrant women is analyzed. The other parameters will be reported separately.

## Results

### Quantitative Data

For analysis, only questionnaires with completed answers for the first 20 questions, the profession and the questions on EDA were included. In the 6 months of data collection between May and October 2017, a total of 121 questionnaires could be included for this analysis. According to a broad estimation of the included 16 obstetrics departments which on average employ 12 obstetricians and 30 midwives (n = 672), this added up to a response rate of 18%.

In the quantitative study, midwives, and obstetricians both saw fears of the women as the most important reason for a lower rate of EDA among immigrant women. Interestingly, the midwives regarded this even more often as the most important reason (85%) compared to the obstetricians (54.3%). The second most given reason was a language barrier, as in this case the patient information is regarded by the respondents as too time consuming. Respondents of both professions think as well that immigrant women desire an EDA less often. Obstetricians think more often (54.3%) than midwives (30%) that immigrant women do not know about the option of an EDA (for details of the quantitative results see Table 1).

**Table 1:** Reasons for lower rate of EDA among immigrant women during labor and birth given by medical staff in obstetric clinics in Berlin (multiple answers may be possible)

Category		Midwives N (% within profession)	Obstetricians N (% within profession)	p value
“Demand”	Less frequently desired	14 (35%)	34 (42%)	0.555
	Unknown as an option	12 (30%)	44 (54.3%)	0.013*
	Uncertainties and fears of the women	34 (85%)	44 (54.3%)	0.001*
“Supply”	Incorrect translation by companion	11 (27.5%)	23 (28.4%)	1
	Legal reservations due to insufficient informed consent	17 (42.5%)	28 (34.6%)	0.428
	Not sufficient information communicable due to language barrier	7 (17.5%)	23 (28.4%)	0.264
	Adequate patient information too time-consuming due to language barrier	16 (40%)	44 (73.3%)	0.177

*Significance level is p ≤ 0.05 in the Fisher’s Exact Test on the two-sided level

### Qualitative Data

#### Qualitative Data from the Online-Survey

Of the respondents (n = 121), 19 gave a free answer in their own words in addition to the multiple choices offered in the online survey. Thematic grouping of the free answers to questions in the online survey confirmed some of the above-mentioned categories, but also brought up new categories.

The “demand” side was reflected also in the free answers. The woman’s fear was mentioned by two respondents. Different aspects were given why EDA is less frequently wanted by immigrant women by nine respondents. While some respondents mention categories such as “tradition” or “culture”, one respondent makes it more specific and explains it the following way: “Painful contractions are accepted as fate and therefore accepted”. Another respondent states, “Sometimes they have other expectations of the birth and the role of the birth attendant”.

The “supply” side in terms of more time-consuming information of the woman is only mentioned by one respondent: “In the case of a language barrier, it is difficult for the woman to explicitly ask for EDA. I have the feeling that it is offered less often [by the health care staff] due to time-consuming patient information.”

What is most interesting in the free answer section is a third category mentioned by six respondents which describes the interference of the companion inhibiting the self-determination of the immigrant woman. Respondents gave answers such as “The husband rejects the EDA” or “culturally grounded lack of self-determination of the woman, especially due to patriarchal founded views on the necessity of an EDA”.

#### Qualitative Data from the Interview-Study

The interviews gave even more insights into the reasons medical staff see for lower rates of EDA among immigrant women during labor and birth. The aspects of “demand”, especially fears on the side of the women are mentioned as well as. The respondents often stress that in their opinion the fears are not grounded in evidence. One midwife expressed: “They do not want this […], they come and say: ‘No, the cousin of my cousin had back pain after an EDA’—and then that’s it for them. ‘That is negative, no, we do not want that’”.

Another reason for lower EDA rates is given in terms of more acceptance of pain during labor and birth. One midwife says for example: “That immigrant women from their family background just accept it like that: birth hurts and I can accept the pain”. This acceptance of pain during labor and birth is expressed by a doctor in the following way: “and maybe they get taught by their mother and all their aunts: well, we all went through this, one will stand this.”

Another aspect of “demand” is the knowledge and familiarity with the options of the German health care system on the part of the immigrant women. One midwife supposes that the degree of acculturation might nihilate the differences between immigrant and non-immigrant women regarding choice for EDA: “It is getting less and less the longer they are here of course, the longer they know a different kind of health care system. This can be seen also by the second generation of immigrants [parents migrated, but patient born in Germany], where this difference stops.”

The aspects of “supply” stressing for example, the reluctance of medical staff to provide detailed information in the case of a language barrier can also be found in the interview material. We observed the argumentation, that the anesthesiologist was not willing to perform EDA in cases of language barrier for fear that consent is not valid with such patients: “it is a matter of informed consent and thus dependent on the anesthesiologist”. Another obstetrician phrased it: “Sometimes for sure they are deprived of this option because of the opportunities of providing adequate information. But we cannot provide a translator at two in the morning. And when the anesthesiologist insists formally that informed consent is performed, then it is dependent on that person. So unfortunately, we [obstetricians] are dependent on the other group of professions [anesthesiologists]”.

But it is not only the anesthesiologists that are worried about the consequences of a language barrier. One midwife phrased it: “When I cannot inform a woman, then I do not do any risky things there.”

As highlighted above in the free answers in the online surveys, additionally the interviews highlight the aspect of loss of self-determination of the immigrant women in the case of language barrier and a dominant companion. One obstetrician said: “Sometimes it is not the women, who did not want to have it, but the relatives that say: ‘No, she doesn’t get an EDA.’ We experience this as well and especially when the woman does not speak German, then it is natural when the husband says that the wife does not get an EDA.” Another obstetrician phrased it this way: “I think it is not due to the more natural take on giving birth but due to family members, who do not want EDA or do not let the woman complete her sentences and insist on their own wishes. We have had that very often. I think this is more relevant than the wishes of the woman.” Midwives mentioned the same ideas, for example in this way: “Even if the woman often wants to have EDA during birth, the companions often know how to prevent that.”

## Discussion

It is known that perception of pain is influenced by acculturation [14] and pain described in affective terms varies among ethnicities [15]. Although a lower rate of EDA during labor and birth among immigrant women has been shown in various settings [5–7], few authors give any reasons for this discrepancy. Petruschke et al. discussed two main clusters of reasons for lower EDA rates among immigrants [8]. One explanation is the ‘‘supply’’: the medical staff might not pay as much attention to the expressions of pain of immigrant women during the delivery or there may be language barriers/communication problems when providing information and explaining about the options for perinatal pain control. Alternatively, the “demand” might be a reason: immigrants seem to demand EDA less often due to lack of knowledge, anxiety about side effects or because of communication problems [5]. The qualitative study by Petruschke et al*.* stresses mostly the “demand”-driven argumentation [8]. However, that study was conducted prior to giving birth, thus the actual process of the decision on EDA during labor and birth under pain including potential language barriers was not investigated. For an overview of the possible reasons for unequal provision/use of EDA among immigrants during labor and birth derived available in the literature see Table 2. Our study gives new insights into this topic.

**Table 2:** Possible reasons for unequal provision/use of EDA among immigrants during labor and birth derived from the literature

Cluster	Reason	Source
Demand	Less frequently desired	[16]
	No knowledge about option	[5]
Supply	Incorrect translation by accompanying person	[17, 18]
	Legal reservations: Informed consent not possible, worry that consent is not valid in the case of a language barrier	[19]
	Lack of opportunity to providing adequate information (for example time constraints)	[20, 21]
Possibly both	Language barrier	[22]

*EDA* epidural anesthesia

It seems as if the reasons for a lower EDA rate during labor and birth among immigrant women in Berlin is multifactorial. Our study supports the significance “supply” and “demand” reasons proposed by Petruschke et al. [8]*.* We give further insights into the reasons behind lower “demand”. The respondents in our study have the impression that the acceptance of pain is higher among immigrant women.

The view of the medical staff conflicts in a way with the data collected in interviews with Turkish immigrant women by Petruschke et al*.* While the latter stress that Turkish women themselves decide about the choice for or against EDA, the medical staff raises the issue of potential barriers to self-determination of immigrant women with limited German proficiency. This hypothesis adds to the categories of reasons proposed by Petruschke et al. [8] with the new category “structural barriers to self-determination”.

A limitation of our study is that all perspectives of mothers and medical staff are given from the respective personal views. We did not conduct interviews with immigrant women themselves nor did we perform participant observation. Immigrant women should be interviewed about their decisions against or for an EDA during labor and birth after giving birth. It could be possible, especially in the case of the aforementioned “structural barriers to self-determination”, that women are not aware of subtle processes in the decision in the delivery room. Furthermore, they might not be willing to admit that they felt oppressed by their husband or other female accompanying persons or they might fear repression if they voice this. Further investigation focusing more on ethnographic data collected through participant observation could contribute valuable insights.

The clinical routine of the medical staff supports the data that immigrant women receive less EDA and we have outlined new reasons here in this report. What is the clinical significance of this? In the study of Yoong et al*.* from the UK, the authors conclude that as EDA prolongs labor, the Kosovo Albanian women have shorter births due to less frequent use of EDA [6]. Furthermore there is data, that continuous care for example through a doula lowers perceived pain during labor [23–26]. We can also see how important it is that the process of giving birth is accompanied by someone who is known to the women in advance by data on how continuity of care through midwives improves satisfaction with care [27].

Clinical observations often show more support by female family members during labor and birth among immigrants than among non-immigrants [28]. The presence of these female members of family might lower the perceived care during labor and birth just as one‐to‐one intrapartum support have proven to do in other studies [29, 30]. Moreover, our results also suggest that female family members that have had their own birth without pain medication might be discouraging or even inhibiting use of pain medication for their relatives.

A qualitative study among Turkish women in Berlin identified medically unfounded fears about possible side effects of a PDA as an impeding factor for EDA use [16]. The question is of course whether the reported fears are so medically unfound. There is some evidence that the rate of chronic back pain is increased after EDA during labor [31]. A more recent review did not support this conclusion though [32]. Furthermore, although the risk of severe neurologic complications in a recent review is evaluated as lower than previously thought, it is not zero [33]. It can be reasonable, thus, to decide against EDA on the grounds of fear of side effects. But this ought to be taken into perspective. The fears health care staff are reporting on in the interviews are often without scientific proof and not put into perspective with data on frequency of specific potential side effects.

The effect of length of labor due to EDA is often debated. Some authors raise the concern that epidural analgesia increases the duration of the second stage of labor by 15 to 30 min and may increase the rate of instrument-assisted vaginal deliveries as well as that of oxytocin administration [34]. A recent review concludes that data regarding the effect of EDA on duration of labor is conflicting [32]. Hence as the question of if EDA leads to longer duration of labor cannot at this moment been finally answered the main argument for or against EDA should be the wish of a woman giving birth to reduce the pain she is feeling.

Although evidence is conflicting and an EDA is not per se a criterion for good care, one of the main foundations of our health system is enough information for the patient in her language so that she can make her own decisions. The lower “supply” as pointed out by Petruschke et al*.* already is a problem [8]. We know from the USA-context that in the case of a language barrier, provision of care is perceived as more stressful and of lower quality [20]. This could also be shown in our recent survey of medical staff in Berlin [11]. The “structural barriers to self-determination” are also very important to highlight. It has been reported, for Latina patients in the USA, that non-professional interpreters convey information more often incorrectly [18]. Language is a key aspect for provision of good care [35]. Especially for women who for a plethora of reasons might not speak German sufficiently, once they are treated within our health system, they need to be provided with good information, and to protect them from any power imbalances that may exist within their families. Widespread access to professional interpreters at any time of the day would be helpful to overcome “structural barriers to self-determination” as well as lower “supply”. The latter, especially, when considering that the lower “supply” is due to concerns about informed consent. Written information in the patient’s native language handed out during birth-preparation courses or general check-ups during pregnancy would be additionally helpful.

## Conclusion

In the view of the medical staff, the reasons for a lower rate of EDA during birth for immigrant women are varied. One side shows that this is attributed to the wishes of the respective women (“demand”) but also could be due to the health care system (“supply”). In the case of a language barrier, the supply, and the access to EDA for immigrant women is limited and the decision regarding an EDA is supposedly shifted to the German-speaking companion (“structural deprivation of self-determination”). We need more structural changes in the German health system for institutionalized translations.
